# LAF: Logic Alignment Free and its application to bacterial genomes classification

**DOI:** 10.1186/s13040-015-0073-1

**Published:** 2015-12-08

**Authors:** Emanuel Weitschek, Fabio Cunial, Giovanni Felici

**Affiliations:** 1 Department of Engineering, Uninettuno International University, Corso Vittorio Emanuele II, 39, Rome, 00186 Italy; 2 Helsinki Institute for Information Technology HIIT, Department of Computer Science, University of Helsinki, P.O. Box 68 (Gustaf Hällströmin katu 2b), Helsinki, FI-00014 Finland; 3 Institute of Systems Analysis and Computer Science “A. Ruberti”, National Research Council, Via dei Taurini 19, Rome, 00185 Italy

**Keywords:** Supervised classification, Alignment-free sequence comparison, Bacterial taxonomy

## Abstract

Alignment-free algorithms can be used to estimate the similarity of biological sequences and hence are often applied to the phylogenetic reconstruction of genomes. Most of these algorithms rely on comparing the frequency of all the distinct substrings of fixed length (*k*-mers) that occur in the analyzed sequences.

In this paper, we present Logic Alignment Free (LAF), a method that combines alignment-free techniques and rule-based classification algorithms in order to assign biological samples to their taxa. This method searches for a minimal subset of *k*-mers whose relative frequencies are used to build classification models as disjunctive-normal-form logic formulas (*if-then rules*).

We apply LAF successfully to the classification of bacterial genomes to their corresponding taxonomy. In particular, we succeed in obtaining reliable classification at different taxonomic levels by extracting a handful of rules, each one based on the frequency of just few *k*-mers.

State of the art methods to adjust the frequency of *k*-mers to the character distribution of the underlying genomes have negligible impact on classification performance, suggesting that the signal of each class is strong and that LAF is effective in identifying it.

## Background

The field of biological sequence analysis relies on mathematical, statistical, and computer science methods for discovering similarities among different organisms, understanding their features and their structure, detecting ancestry, relatedness, evolution, and common functions.

Several well-established sequence comparison algorithms are based on sequence alignment: they compute sequence similarity by aligning portions of sequences (e.g., subsequences) that have common nucleotide assignments. The alignments of two or more sequences are scored according to the number of common nucleotides. Such methods can be exact or heuristic. Among exact methods, Smith-Waterman [1] and Needleman-Wunsch [2] use dynamic programming techniques. The first performs local sequence alignment: it detects the common regions between two sequences by comparing segments of all possible lengths. The second is a global alignment algorithm, designed to align entire sequences. In order to reduce the computational burden of exact methods, several heuristic algorithms have been designed, the most renowned being FASTA [3] and BLAST [4]. For the comparisons of more than two sequences, there are ad-hoc algorithms like Muscle [5], ClustalW [6], Motalign [7], and Mafft [8]. Alignment-based sequence analysis algorithms have a very high computational cost, especially when applied to a large set of sequences [9]. Other problems may also be encountered when performing alignment on genome sequences, related with the presence of non-coding subsequences, or simply with the computational burden associated with the alignment of whole genomes [10].

In order to address these issues, alignment-free sequence analysis methods can be considered. Such algorithms are mainly classified in two groups: methods based on sequence compression and methods that rely on the frequencies of the subsequences (oligomers) [9].

The first class of methods compute a model that succinctly describes the sequence, and assess the similarity of the sequences by analyzing their compressed representations, e.g., Kolomogorov complexity [11] or Universal Sequence Maps [12].

In this work we focus on the second class of methods, alignment-free algorithms that rely on oligomer frequencies and map two strings *X* and *Y* onto corresponding multidimensional vectors **X** and **Y**; these vectors are indexed by a number of substrings in the given alphabet (a typical case is when all possible substrings of a predefined length *k* are used). **X**[*W*] and **Y**[*W*] – the element of **X** and **Y** associated with substring *W* – contain the number of occurrences of *W* in *X* and *Y* respectively. Often the number of occurrences is normalized and converted into a measure of statistical surprise using the length and distribution of characters in each string. Standard distance functions on vectors are then applied to **X** and **Y**, allowing the original strings to be compared by classical distance-based algorithms.

Alignment-free algorithms are currently the most scalable class of methods for reconstructing phylogenetic trees from thousands of large, distantly-related genomes and proteomes [13, 14].

The success of alignment-free methods rests on extensive information on the substring composition of genomes and on codon-usage biases, cumulated over approximately fifty years, with particular emphasis on prokaryotes: from the first studies of GC content [15], to the first detection of biases in the composition of pairs and quadruples of adjacent nucleotides [15–21], to the discovery of species-specific frequencies of 4-mers and 8-mers preserved in DNA fragments ranging from 40 kilobases to 400 bases [22–26], to more recent, unsupervised classifications [27–29] and more complex protein motifs [30].

Since the very beginning, most such studies have relied on some form of noise filtration, either assuming an independent and identically distributed source or a Markov source of low order (i.e., normalizing the raw frequencies using their expectation and or variance according to the specified sources). Markov chains inferred from genomes have indeed been shown to reproduce large fractions of the frequency distribution of *k*-mers in the original genomes [23, 31, 32].

So far, classification has always relied on the frequency of *allk*-mers [27, 33], and minimality in phylogenetic signal has been investigated with respect to the length of the strings from which *k*-mers are extracted, rather than to the space of features used for classification. This trend continues in modern applications of *k*-mer composition to annotating and binning metagenomic reads [34]: increasingly more sophisticated heuristics have allowed to reliably classify reads ranging from one kilobase to 75 bases, under a variety of species abundance scenarios [35–40]. However, fundamental questions on the distribution and concentration of phylogenetic signal in the space of all *k*-mers are still open and scarcely investigated. Among the few attempts in this direction, we mention the use of singular value decomposition (SVD [41, 42]) and of irredundant shared substrings [43] in phylogeny reconstruction, the use of few selected *k*-mers in barcoding genes [44], and early attempts at classifying protein families using the frequency of a small set of dipeptides [45].

In this work, we search for *a minimal set of k-mers whose frequency is sufficient to classify entire genomes*. Specifically, we focus on *logic formulas* (*if-then rules*) whose attributes *W* are *k*-mers, and whose values *f*_*X*_(*W*) are relative frequencies in a genome *X*, possibly corrected by expected counts. An example of such a formula could be:

$$\text{if}\,\,(f(\texttt{ACGT})>0.15) \wedge (f(\texttt{GGCT})<0.6)\,\,\text{then}\,\,X \in \mathcal{T} $$

where $\mathcal {T}$ is a taxonomic unit (for example, *E. coli*) at a given taxonomic rank (for example, at the species level). Similar to recent DNA barcoding efforts, such formulas approximate a unique signature of set $\mathcal {T}$, but they work on entire genomes rather than on few specific genes, and they do not require $\mathcal {T}$ to be at the species level [44, 46]. Contrary to *markers* [47–49], the *k*-mers in such formulas need not to be genes, they need not to be rare in the genomes they characterize, they need not to be absent from the genomes they do not characterize. Contrary to *discriminating substrings* (see e.g. [50] and references therein), formulas can use multiple substrings to classify, and they can link frequencies with conjunctions and disjunctions.

In this paper, we experiment with four rule-based algorithms [51] that extract classification models in the form of logic formulas and we compare them with other state-of-the-art classifiers, such as Support Vector Machines [58, 69] and Nearest Neighbor [70]. Surprisingly, it turns out that we can reliably classify genomes at multiple taxonomic levels using a limited number of formulas, each involving few, short *k*-mers. Moreover, standard noise filtration methods have minimum impact on classification performance, suggesting that noise is automatically dampened by the formula-extraction algorithms.

## Methods

In this section, we present the *Logic Alignment Free* (LAF) technique and software package. The aim of LAF is to classify biological sequences and assign them to their taxonomic unit with the aid of a supervised machine learning paradigm [51] (see subsection *Supervised machine learning and rule-based classification algorithms* for more details). LAF uses a feature vector representation of the biological sequences, and gives them as input to rule-based classification algorithms (for a detailed analysis of rule-based classification methods, see [52]).

In [53], LAF has been already successfully applied to the classification of selectively constrained DNA elements, which are not alignable and do not come from the same gene regions.

Conversely, here we present the method in detail, provide the scripts and the software, and describe its application to bacterial genomes. In the following subsections, we illustrate the feature vector representation technique, the rule-based classification algorithms, and their integration in the LAF framework.

### Representing the sequences as feature vectors with alignment-free methods

The most widespread alignment-free methods compute the frequencies of the substrings in the biological sequences, called *k*-mers (where *k* is the length of the substring). For each sequence, the substring frequencies are then represented in a vector, called frequency vector [12, 54–57]. Each element of this vector expresses the frequency of a given *k*-mer, computed by scanning a sliding window of length *k* over the sequence.

More formally [9], let *S* be a sequence of *n* characters over an alphabet *Σ*, e.g. *Σ*={*A*,*C*,*G*,*T*}, and let *k*∈ [ 1…*n*]. If *K* is a generic substring of *S* of length *k*, *K* is called a *k*-mer. Let the set *V*={*K*_1_,*K*_2_,…,*K*_*m*_} be all possible *k*-mers over *Σ*, and define *m*=|*Σ*|^*k*^ to be the size of set *V*. The *k*-mers are computed by counting the occurrences of the substrings in *S* with a sliding window of length *k* over *S*, starting at position 1 and ending at position *n*−*k*+1. A vector *F* contains for each *k*-mer the corresponding counts *F*=*c*_1_,*c*_2_,…,*c*_*m*_. The frequencies are then computed accordingly and stored in a vector *F*^′^=*f*_1_,*f*_2_,…,*f*_*m*_; for a *k*-mer *K*_*i*_, the frequency is defined as $f_{i}=\frac {c_{i}}{n-k+1}$.

These numerical representations of the sequences allow the use of statistical and mathematical techniques; indeed, the most used approach for sequence comparisons in alignment-free vector representations are distance measures, such as the Euclidean distance and the *d*2 distance [9]. While the authors of [56] use feature vector representation in combination with supervised machine learning methods, specifically Support Vector Machines [69] for biological and text sequences, here we propose to analyze the frequency vectors with rule-based supervised machine learning algorithms. The effectiveness of this technique is investigated and tested on bacterial sequences.

### Supervised machine learning and rule-based classification algorithms

The aim of this step is to classify the biological sequences into their taxonomic unit. Once the sequences are represented in a vector space, it is possible to analyze them by adopting a supervised machine learning approach, sketched in the following.

Given a set *B* of biological sequences, each assigned to a taxon (*training set*), a classifier is trained with these sequences in order to compute a classification model that predicts the taxon of each sequence from the values of its vector space representation. An additional set of sequences with known taxa is used to evaluate whether the model computed on the training set is able to predict correctly the taxa (the latter is called *test set*). For assessing the performance of the classifier we adopt the accuracy measure (A), also called correct rate $A=\frac {c}{t}$, where *c* is the number of correct classified sequences in the test set and *t* is the number of total sequences in the test set.

We focus on a particular type of classification methods - rule-based classifiers - which express the classification model in propositional logic form (e.g., *if-then rules*). Rule-based classifiers have the main advantage of being able to control their dimension (in this case, the number of *k*-mers used), they are easily interpretable, and can straight-forwardly be integrated with other contextual knowledge. Several rule-based classification methods are proposed in the literature; in LAF we adopt the following ones: Data Mining Big (DMB) [59, 60], RIDOR [61], PART [62], and RIPPER [63]. All these methods use distinct rule extraction approaches, but – as we will see later – perform very well on the analyzed data sets of bacterial sequences. We report a brief description of these methods in the following.

**Data Mining Big** (DMB) [60, 64, 65] is a rule-based classification software designed for biomedical data. It adopts optimization models that are formulated and solved in order to deal with the different steps of the data mining process. Five main steps are performed by DMB:

1. discretization: conversion of numeric attributes into nominal (discrete);
2. discrete cluster analysis: samples that are similar in the discretized space are clustered and dimension-reduced accordingly;
3. feature selection: the most relevant attributes for classification purpose are selected;
4. rule extraction: small and effective rules are extracted from training data and verified on test data;
5. classification: the extracted rules are used to classify new samples.

**RIDOR**[61] performs rule extraction directly from the training data set. The first step is the computation of a default rule for the most frequent class (e.g., “all sequences are E. coli”). Then, it computes exception rules that represent the other classes (e.g., “except if *freq*(*ACGT*)<0.45 then the sequences are S. aureus”).

**PART** [62] performs rules extraction with an indirect method. It uses the C4.5 decision tree based classification algorithm [66], which computes a pruned decision tree for a given number of iterations. The best performing tree in terms of classification performances is chosen by PART and converted to rules for every species.

**RIPPER** [63] is a direct rule extraction method based on a pruning procedure, whose aim is to minimize the error on the training set; it performs the following steps: i) growth of the rules; ii) pruning of the rules; iii) optimization of the model; iv) selection of the model. In the first step, thanks to a greedy procedure, RIPPER extracts many classification rules. Then, the rules are simplified and optimized in step two and three, respectively. Finally, the best model (i.e., set of rules) is selected.

### Logic Alignment Free (LAF) method

Rule-based classifiers have been successfully used in the analysis of aligned sequences, e.g., in [59] and [60], where the classification of biological sequences to their species is performed by considering only sequences from the same gene region. In this case the rule-extraction procedure identifies exact gene regions and nucleotide assignments that are specific to a species; an example of such a rule could be ‘’if pos354 = T of gene 16S then the sequence belongs to E. Coli”.

Here we test a method for classifying biological sequences without the strict requirement of overlapping gene regions and of calculating an alignment, referred to as Logic Alignment Free (LAF). It is based on the frequency vector representation of the sequences. The method allows to classify non coding DNA that is not alignable [53], and whole genomes, whose alignments are very computationally demanding. LAF adopts a supervised machine learning procedure, where a labeled training set of whole genomes is considered (labels in this case would be associated to the taxon). LAF would then operate with the following steps, if we take into account every genome *g* of the input data set:

- The genome *g* is reverse complemented, the *k*-mers with *k*∈ [ 3…6] are counted and stored in a frequency vector *F*^′^;
- A matrix that contains all frequency vectors is created; the rows of the matrix are associated to the *k*-mers and the columns to the sequences (an example is given in Table 1);

**Table 1:** Example of frequencies vectors matrix extracted by LAF and provided as input to rule-based classifiers

	*S* *e* *q*_1_	*S* *e* *q*_2_	…	*S* *e* *q*_*n*−1_	*S* *e* *q*_*n*_	
	*E. Coli*	*E. Coli*	…	*S. Aureus*	*S. Aureus*	
AAA	0.46	0.26	…	0.24	0.26	
AAC	0.12	0.16	…	0.23	0.24	
AAG	0.13	0.23	…	0.23	0.22	
…	…	…	…	…	…
- The frequencies are discretized with the MDL procedure [67] before applying RIDOR, PART and RIPPER, while DMB provides its own built-in discretization method;
- A set of four rule-based classifiers (e.g., DMB, RIDOR, PART and RIPPER) take the matrix as input and extract the classification models and specimen to taxonomic unit assignments;
- The above is repeated for different combinations of training / test sets.

**Fig. 1:**
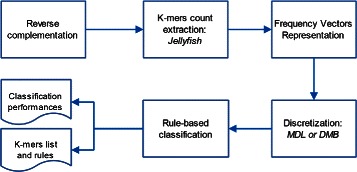
Flow chart of the LAF method

For a compact overview of the method the reader may refer to the LAF flow chart drawn in Fig. 1. To compute *k*-mer counts, we adopt the Jellyfish software [68]. Data discretization is performed using MDL [67] or the DMB internal procedure. As rule-based classifiers implementations we employ the Weka [67] and the DMB packages. The LAF method is deployed in a software package available at dmb.iasi.cnr.it/laf.php.

### Data sets of bacterial genomes

In order to prove the validity of the LAF technique, we chose to test the method for the classification of biological sequences belonging to the bactria domain. We downloaded 1964 bacterial genomes from the NCBI genomes database (www.ncbi.nlm.nih.gov/genome/browse/). For every downloaded sequence, we query the NCBI taxonomy service (scripts are available at dmb.iasi.cnr.it/laf.php) to retrieve the full lineage, i.e., Species, Genus, Order, Class, Phylum. In order to perform an effective classification, we do not take into consideration under-represented species and therefore we filter out sequences with less than nine specimens. This step is necessary to perform a proper training of the classifiers. The final *filtered data set* is composed of 413 sequences with 25 species, 21 genera, 14 orders, 9 classes, and 6 phyla. Additionally, we also report the performances on the *original data set* (1964 bacterial genomes, 1157 species, 590 genera, 120 orders, 57 classes, and 36 phyla).

## Results and discussion

We apply LAF to the previously described *filtered data set* of bacterial genomes, setting *k*∈ [ 3…6] and using the four already mentioned rule-based classification algorithms by adopting a 10-fold cross validation sampling scheme. We show also the results on the *original data set* composed of 1964 sequences. Additionally, we compare the results of LAF with respect to the Support Vector Machine (SVM) classifier [69] and with respect to a Nearest Neighbor approach [70].

**Fig. 2:**
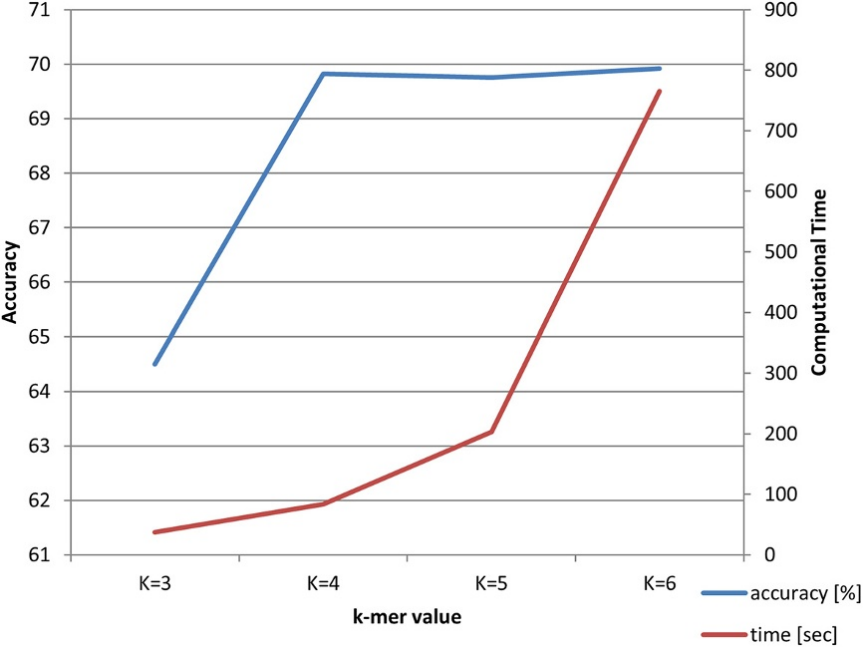
Accuracy and computational times of RIPPER with respect to increasing values of *k* on the original data set

First, we test LAF on the filtered raw sequences without any preprocessing, obtaining very good classification performance. The accuracy of the classification algorithms for *k*=4 and multiple taxonomic levels is summarized in Table 2. We focus on *k*=4 here since it is the smallest value to achieve good classification performances: increasing *k* slightly improves classification performances, but also complexity and computational time. We justify the choice of *k*=4 providing experimental evidence in Table 3 by focusing on the order level since similar performance is obtained at other levels. We can see that the classification accuracy only slightly increases by raising the value of *k*, but complexity and computational time significantly do. We provide also an example in Fig. 2 that shows the accuracy and computational time of RIPPER with respect to increasing values of *k*. The *k*-mers extraction is linear in the size of the input, but it is worth noting that for greater values of *k* the required IO bandwidth and the size of the data matrices exponentially increase [68], slowing down the *k*-mers extraction and the classification processes. Additionally, the value of *k*=4 resonates with a number of previous studies [71–73].

**Table 2:** Percent accuracy of the rule-based classifiers for each taxonomic unit (10-fold cross validation) on the filtered data set

Level	RIPPER	RIDOR	PART	DMB	Avg ±std.dev
Species	93.21	97.33	96.36	**97.61**	96.13 ±2.0
Genus	93.98	**98.79**	97.10	98.44	97.08 ±2.2
Order	98.79	**99.27**	98.31	98.58	98.74 ±0.4
Class	96.50	97.81	**98.79**	97.06	97.79 ±0.9
Phylum	96.88	**98.78**	98.07	98.53	98.06 ±0.8
**Avg** ±**std.dev**	95.87 ±2.2	**98.40** ±0.8	97.72 ±1.0	98.24 ±0.4	97.55 ±1.0

The best performances are highlighted in bold for each taxon

**Table 3:** Accuracy (ACC) [%] and computational times (T) [sec] on the order level with different values of *K*

Data set	Classifier	K=3		K=4		K=5		K=6	
		ACC	T	ACC	T [s]	ACC	T	ACC	T
Original	RIPPER	64.50	*37.08*	69.82	83.53	69.76	203.53	69.92	765.34
Original	RIDOR	61.63	*71.17*	62.25	320.72	64.19	1509.75	64.75	10320.40
Original	PART	65.37	*12.67*	67.05	24.58	67.77	70.13	70.02	280.23
Original	SVM	70.69	*605.55*	85.37	937.32	88.59	1312.52	89.56	2020.60
Original	NN	83.27	*9.56*	85.67	12.13	86.49	19.34	87.06	114.48
Filtered	RIPPER	98.79	*0.82*	98.79	1.55	99.27	4.56	98.79	27.76
Filtered	RIDOR	96.12	*1.58*	99.27	3.05	96.36	26.16	97.33	34.31
Filtered	PART	97.34	*0.51*	98.31	1.00	97.58	2.28	97.09	23.11
Filtered	SVM	99.56	*10.62*	99.87	11.58	99.65	13.10	99.68	14.71
Filtered	NN	99.45	*1.99*	99.93	3.30	99.34	3.70	99.63	4.18
**Average**	-	83.67	*75.2*	86.63	139.88	86.90	316.51	87.38	1360.51

In Table 2, we report the average accuracy over all classification algorithms on the *filtered data set*. We note that the best results (98 % accuracy) are obtained for the phylum level – the highest in the taxonomy. Accuracy remains greater than 96 % at lower levels as well. According to the average over all taxonomic levels, RIDOR exhibits the best performance.

Moreover, we compare LAF with respect to the Support Vector Machine (SVM) classifier. We adopt the Weka implementation of SVM (called SMO) with a linear kernel and a soft margin. We obtain an accuracy of 99 % on the filtered data sets with a 10-fold cross validation sampling scheme, which slightly outperforms LAF. But we remark that SVM outputs just a single classification model that cannot be easily interpreted by human experts.

Finally, we evaluate also the performances of the *Nearest Neighbour* (NN) classifier by using the Weka implementation of NN (called IBk) and by setting the number of neighbours to 1, the NN search algorithm to linear, and by adopting the Euclidean distance. Also in this case we obtain an accuracy of 99 % on all filtered data sets with a 10-fold cross validation sampling scheme, but no human readable classification model.

Conversely to NN and SVM, the rule-based classification methods adopted by LAF provide sets of similar rules than can be analyzed, compared, and evaluated by the user. Here we consider as a sample the rules at the species level extracted by DMB, reported in Table 4. A representative example of such family of rules is the one for *Helicobacter pylori*: ”if 5.56≤*f*(GTAC)<42.82 then the sample is *Helicobacter pylori*”. Here *f*(*K*) is the frequency of substring *K* (for readability, the frequency values are multiplied by 10^5^).

**Table 4:** A sample of classification rules at the species level extracted by the DMB software. *f*(*W*) represents the relative frequency of substring *W* in a genome, multiplied by 10^5^ for readability

A. baumannii	*f*(GTAC)≥229.10∧*f*(TGCA)≥515.63
B. cereus	384.04≤*f*(CTCA)<490.11∧819.04≤*f*(TCCA)<875.80
B. animalis	762.28≤*f*(TCCA)<819.04∧469.35≤*f*(TGCA)<515.63
B. longum	*f*(GTAC)≥229.10∧330.52≤*f*(TGCA)<376.80
B. aphidicola	57.77≤*f*(AGGC)<182.81
C. jejuni	490.11≤*f*(CTCA)<596.17∧353.97≤*f*(CTGA)<451.85
C. trachomatis	305.55≤*f*(GGAC)<393.10∧875.80≤*f*(TCCA)<932.56
C. botulinum	371.77≤*f*(ACTC)<434.37∧112.00≤*f*(GCAC)<261.71
C. diphtheriae	819.04≤*f*(TCCA)<875.80∧423.07≤*f*(TGCA)<469.35
C. pseudotuberculosis	875.80≤*f*(TCCA)<932.56∧423.07≤*f*(TGCA)<469.35
E. coli	710.86≤*f*(GCAC)<860.58∧415.84≤*f*(GCTA)<525.98
F. tularensis	592.00≤*f*(TCCA)<648.76∧330.52≤*f*(TGCA)<376.80
H. influenzae	549.73≤*f*(CTGA)<647.60∧130.47≤*f*(GGAC)<218.01
H. pylori	5.56≤*f*(GTAC)<42.82
L. monocytogenes	411.43≤*f*(GCAC)<561.15∧305.55≤*f*(GGAC)<393.10
M. tuberculosis	649.71≤*f*(ATCA)<772.78
N. meningitidis	590.29≤*f*(GATA)<754.27∧376.80≤*f*(TGCA)<423.07
P. marinus	(*f*(AGGA)<602.46∨*f*(AGGA)≥706.28)∧*f*(GCTA)<856.37
	∧117.33≤*f*(GTAC)<154.58
S. enterica	525.98≤*f*(GCTA)<636.11∧393.10≤*f*(GGAC)<480.64
S. aureus	1082.23≤*f*(GATA)<1246.22∧*f*(GTAC)≥229.10
S. pneumoniae	393.10≤*f*(GGAC)<480.64∧154.58≤*f*(GTAC)<191.84
S. pyogenes	596.06≤*f*(AGTA)<733.86∧1082.23≤*f*(GATA)<1246.22
S. suis	918.25≤*f*(GATA)<1082.23∧330.52≤*f*(TGCA)<376.80
S. islandicus	218.01≤*f*(GGAC)<305.55∧284.24≤*f*(TGCA)<330.52
Y. pestis	596.17≤*f*(CTCA)<702.24∧*f*(CTGA)≥941.24

We observe that the same 4-mer is able to distinguish 3 and 2 bacterial species with different frequency values, respectively, and that twenty 4-mers suffice to separate all the 25 species. The classification rules are also very concise, since most of them are composed only by the conjuction of the conditions on two 4-mers (in the logic jargon, such rules are conjunctive clauses composed of two literals). In general, the rules computed for distinct species do not seem to use disjoint, species-specific sets of *k*-mers, suggesting that discrimination critically depends on the frequency of a *k*-mer rather than on its simple presence or absence in a species. Additional considerations derive from the granularity of the adopted discretization. The method allows to specify up-front the number of intervals used to discretize the frequency values of each *k*-mer, and then searches for an optimal discretization under this condition. From the experimental results we conclude that the number of intervals in which frequencies are discretized has minimal effects on classification quality, provided that at least 3 intervals are used (results not reported).

Moreover, we show the results on the *original data set* of all rule-based algorithms and compare them with SVM and NN in Table 5. It is worth noting that the methods are not able to classify the bacteria genomes at species level, because of under representation (i.e., there are many species with just one or two sequences). At higher taxonomic levels (class and phylum) we obtain more reliable results. We highlight that SVM and NN perform best, but they do not provide a human readable classification model as rule-based classifiers, which permit to identify the different taxon specific *k*-mers.

**Table 5:** Percent accuracy of the classifiers for each taxonomic unit (10-fold cross validation) on the original data set

Level	RIPPER	RIDOR	PART	DMB	SVM	NN	Avg ±std.dev	
Species	-	-	-	-	-	-	-	
Genus	54.17	47.67	50.17	48.54	-	**73.04**	45.60 ±24.2	
Order	69.82	62.25	67.05	63.78	85.37	**85.68**	72.32 ±10.5	
Class	75.08	69.92	71.76	72.05	88.43	**89.10**	77.72 ±8.7	
Phylum	75.85	70.99	56.77	71.45	85.93	**86.08**	74.51 ±8.2	
**Avg** ±**std.dev**	68.73 ±10.0	62.71 ±10.7	61.44 ±9.7	63.96 ±11	64.93 ±43.3	**83.48** ±7.1	67.54 ±14.8	

The best performances are highlighted in bold for each taxon

In order to test their effect on the classification performance, we applied different types of preprocessing to the filtered data set suggested in previous works [74–77] about phylogenetic reconstructions of genomes with alignment-free algorithms.

- The first type consists in excluding all high-frequency and low-complexity substrings [74] of a genome from its *k*-mer counts, using the DUST software implementation provided by NCBI [78];
- A second type of preprocessing consists in replacing the frequency *f*_*T*_(*W*) of a *k*-mer *W* in a string *T* with a measure of the *statistical significance* of the event that *W* has *f*_*T*_(*W*) occurrences in *T*. Specifically, we assigned to a *k*-mer *W* the score $z_{T}(W) = \left (p_{T}(W)-\tilde {p}_{T}(W)\right)\!/\tilde {p}_{T}(W)$, where *p*_*T*_(*W*)=*f*_*T*_(*W*)/(|*T*|−*k*+1), and where $\tilde {p}_{T}(W) = p_{T}(W[\!1..k-1]) \cdot p_{T}(W[\!2..k]) / p_{T}(W[\!2..k-1])$ is the expected value of *p*_*T*_(*W*) under the assumption that *T* was generated by a Markov process of order *k*−2 or smaller. This score has been shown to be critical in building accurate phylogenies of distantly-related prokaryotes [75];
- We experimented with the estimator $\tilde {p}_{T}(W) = \left (f_{T}(W[\!1]) \cdot f_{T}(W[\!2..k]) + f_{T}(W[\!1..k-1]) \cdot f_{T}(W[\!k]) \right)/2$, derived under the assumption that *W*[ 2..*k*−1], *W*[ 1] and *W*[ *k*] occur independently in *T* [76];
- We also adopted an even simpler estimator, based on single-nucleotide frequencies (see [9, 77] and references therein for alternative ways to compute $\tilde {p}_{T}(W)$).

In our experiments, none of these preprocessing methods yielded a visible improvement on classification quality, suggesting that noise is automatically dampened by the formula-extraction algorithms run on raw frequencies. Nonetheless, we include in our LAF package an implementation of all such filters, since they could be useful in other data sets.

## Conclusions and future work

The LAF method combines *k*-mer composition vectors and rule-based classification algorithms to classify biological sequences. Such sequences do not need to be aligned or to belong to the same gene. The method was applied to bacterial whole genomes, and it was able to perform with accurate classification results and to identify common subsequences (*k*-mers) in each taxon (class) of the data set.

We compared our method with other state-of-the art classification methods and provided experimental results that show promising performance of LAF in particular in the classification model extraction (i.e., specific *k*-mers for each taxon).

Several directions for future research stem from the results obtained in this paper: further reducing the size of the classification models, analyzing more deeply the *k*-mers selected by our models; and measuring how classification performance degenerates when moving from whole genomes to short fragments.

Another possible way to further reduce the size of our models consists in building *hierarchical* classification rules by extracting logic formulas that best discriminate between elements in a taxonomic unit $\mathcal {T}$ and elements in $\text {\texttt {parent}}(\mathcal {T}) \backslash \mathcal {T}$, where $\text {\texttt {parent}}(\mathcal {T})$ is the parent of $\mathcal {T}$ in the taxonomic tree. Such result would look very similar to a decision tree, and the corresponding *k*-mers could be related to the notion of *crowns* (see [79]).

Analyzing the actual *k*-mers selected by our models is another obvious open direction, for example in terms of syntactic similarity and positional correlations between the *k*-mers that appear in the same formula, or in terms of enrichment of such *k*-mers in regulatory regions or in gene families devoted to specific cellular processes.

It is also of interest the understanding of how the classification performance degenerates when moving from whole genomes to short fragments, for example by determining how small a fragment we can classify correctly using the formulas learned from entire genomes, or using new formulas learned from fragments. *Abundance estimation* in metagenomic samples is also a natural application for the strong biases in the relative frequency of *k*-mers that we report here: given a set of observed *k*-mer frequencies in a sample, and a set of logic rules in sequenced genomes, the problem would then amount to compute the most probable abundance of known species in the sample.
